# Heart Failure Is Closely Associated With the Expression Characteristics of Type I Interferon‐Related Genes

**DOI:** 10.1002/clc.70063

**Published:** 2024-12-20

**Authors:** Jianfeng Zhuo, Yan Zhong, Xiaojuan Luo, Sijie Qiu, Xinmei Li, Yunyu Liang, Yu Wu, Xiyu Zhang

**Affiliations:** ^1^ Department of Geriatrics The Second Affiliated Hospital of Guangzhou University of Chinese Medicine Guangzhou Guangdong China; ^2^ Department of Endocrinology The Second Affiliated Hospital of Guangzhou University of Chinese Medicine Guangzhou Guangdong China

**Keywords:** dilated cardiomyopathy, ejection fraction reduced heart failure, ischemic cardiomyopathy, type I interferon related genes

## Abstract

**Background:**

The association between the expression of type I interferon related genes (TIIRGs) and EFrHF is not well understood. This study aimed to investigate the correlation between the expression patterns of TIIRGs and EFrHF using bioinformatics analysis.

**Materials and Methods:**

An analysis was conducted to examine the expression and distribution of TIIRGs in cardiomyocytes. Afterwards, GSE5406 was utilized as the validation set, including 16 without heart failure, 86 with idiopathic dilated cardiomyopathy (IDCM), and 108 individuals with ischemic cardiomyopathy (ICM). We conducted a comparative analysis of the variations in TIIRGs gene expression across various forms of heart failure.

**Results:**

There were eight genes that showed substantial changes between patients with EFrHF and those without heart failure. A risk model for EFrHF was developed utilizing JAK1 and EIF2AK2, with an area under the curve (AUC) of 0.909. Five genes exhibited notable disparities between IDCM and ICM. Through multivariate analysis, it was shown that JAK1 and IFNA16/IFNA14 were identified as independent risk variables for distinguishing between the two pathogenic categories. The model, utilizing JAK1 and IFNA16/IFNA14, successfully differentiated between IDCM and ICM with an area under the curve (AUC) of 0.722. In the validation set GSE5406, the expression of JAK1 was dramatically downregulated, while EIF2AK2 was significantly upregulated in heart failure (HF) tissues. The model utilizing JAK1 and EIF2AK2 successfully differentiated between those with an illness and those without (AUC = 0.877).

**Conclusions:**

The expression of TIIRGs is strongly associated with the presence and specific subtypes of HF in a pathological context.

## Graphical abstract

Heart failure (HF) is a complex clinical condition characterized by altered gene expression. The expression of Toll‐like receptor‐interacting proteins (TIIRGs) is strongly associated with the presence and specific subtypes of HF in a pathological context.

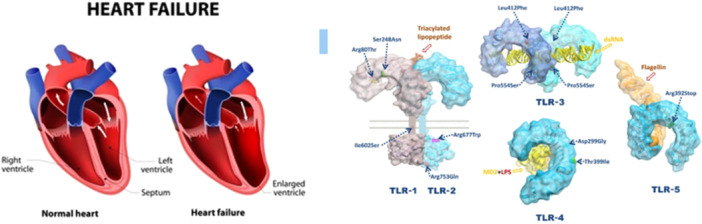

## 1 Introduction

Ejection fraction reduced heart failure (EFrHF) is a collection of disorders characterized by dyspnea and exhaustion as common clinical features. The pathophysiological foundation of this condition is the presence of aberrant cardiac structure and function, resulting from many causes. This leads to dysfunction in both the systolic and diastolic phases of the cardiac cycle, eventually resulting in a reduction in cardiac output and an increase in ventricular filling pressure [1]. EFrHF is the terminal phase of most cardiac conditions, with a high risk of death, which is considered one of the primary threat to human health [2].

According to the data published in the Journal of the American Heart Association in 2014, approximately 26 million patients have heart failure (HF) worldwide, 5.7 million in the United States alone, 670 000 new cases annually [3], and approximately 15 million patients have HF in Europe. In developed countries, the overall HF prevalence rate is 1%–2%, of which the incidence rate of HF in individuals aged ≥ 70 years is > 10% [2, 4]. The overall HF prevalence rate in Asia is similar to that in Western countries, ranging from 1% to 1.3% [5]. Based on epidemiological data in recent years, the HF prevalence in the population aged 35–74 years in China in 2000 was 0.9% (0.7% for males and 1.0% for females) [6].

Ischemic cardiomyopathy (ICM) and dilated cardiomyopathy both are common reasons of EFrHF with difficulty in differentiating them in the late stage of HF. At this time, no obvious difference in clinical performance, ultrasonic examination, and other aspects of ICM and dilated cardiomyopathy is noted between ICM and DCM, and the differential diagnosis often needs percutaneous coronary angiography [7]. Type I interferon (IFN) is a broad‐spectrum antiviral molecule that plays a significant role in the innate immune response to virus. Previous experiments showed that type I IFN can not only induce tumor cell apoptosis but also induce normal tissue cell apoptosis [8], especially in ischemic and nonischemic cardiomyopathy, as well as in viral myocarditis [9, 10, 11]. Previous studies also proved that type I IFN signaling pathway was involved in heart failure [12, 13]. However, the role of type I IFN in the discrimination of ICM and DCN remains unclear.

### 1.1 Objectives

The objective of this study was to investigate the correlation between the expression patterns of TIIRGs and EFrHF through the use of bioinformatics analysis.

## 2 Methods

### 2.1 Study Design

This is a bioinformatic analysis based on open database.

### 2.2 Gene Sets

Using GEO's expression profiling by array data of heart tissues (GSE57345 encompassed a total of 313 cases, including 136 and 177 cases of non‐HF and EFrHF, respectively) as the discovery set, combined with the given clinical characteristics, TIIRG expression and distribution in myocardial cells were analyzed [14, 15]. Subsequently, GSE5406 was used as the validation set, including 210 cases: 16 patients with non‐HF and 194 patients with EFrHF, 86 of whom had idiopathic dilated cardiomyopathy (IDCM) and 108 had ICM [16]. All data were obtained from the published studies and download from the GEO database (ncbi.nlm.nih.gov/geo) [14, 16]. The TIIRGs data set comes from the KEGG database (type I IFN signaling path, N00150), with a total of 31 genes: IFNA1, IFNA2, IFNA4, IFNA5, IFNA6, IFNA7, IFNA8, IFNA10, IFNA13, IFNA14, IFNA16, IFNA17, IFNA21, IFNB1, IFNAR1, IFNAR2, JAK1, TYK2, STAT1, STAT2, IRF9, IRF7, and EIF2AK2. This study was conducted according to the Declaration of Helsinki.

### 2.3 Bioinformatics

Hierarchical clustering analysis was performed on the TIIRGs expressed in the heart tissue of each patient, and group patients with similar gene expression patterns. The differences in TIIRG expression between different groups were determined from the entire data set and grouped using a hierarchical clustering algorithm in the Gene Cluster 3.0 program. To generate heatmaps of cluster indications and HF patterns, the Java TreeView program was used. Gene data were annotated on the GPL11532 platform ([HuGene‐1_1st] Affymetrix Human Gene 1.1 ST Array [transcript version]), and most genes had unique probe IDs. As the positions of two genes on the genome were overlapping and the probe sequence was derived from the overlapping region of these two genes, a one‐to‐many relationship was shown between the probe and SYMBOL. During analysis, these genes with combined probe IDs were not distinguished. When multiple probes corresponded to a gene, the average value was taken for subsequent calculations.

### 2.4 Statistical Analysis

Statistical Package for the Social Sciences version 22.0 (IBM Inc., Armonk, NY, USA) was used for statistical analyzes. To analyze the correlation between clinical features and variables, Fisher's exact test and Pearson correlation analysis were used. Gene expression was compared between different groups using analysis of variance. In GSEA, to screen out the most likely scientifically meaningful hypotheses and reduce the risk of false positives, gene sets with FDR < 0.25 were used as significantly enriched gene sets. *p* < 0.05 was considered statistically significant. We divided all the samples into two sets according to disease and nondisease, and then used logistic regression analysis FSTEP (LR) method to construct a heart failure status prediction model for the variables of 8 differentially expressed genes, with a decision cutoff value of 0.5. We compared the predictive performance of different models by plotting ROC curves and calculating AUC. The model with the least number of predictor variables among the models with a prediction accuracy greater than 0.85 was considered the optimal result. In the models constructed by JAK1 and EIF2AK2, the formula was finally obtained based on the coefficients of the two parameters. After substituting the expression data of different samples into the formula, the risk score could be obtained. This score was used as the prediction result to compare with heart failure status. According to the maximization of sensitivity + specificity, we got cut off value of 0, and AUC of 0.909.

## 3 Results

### 3.1 Comparison of Groups by TIIRG Expression Correlation (Hierarchical Clustering)

**Figure 1:**
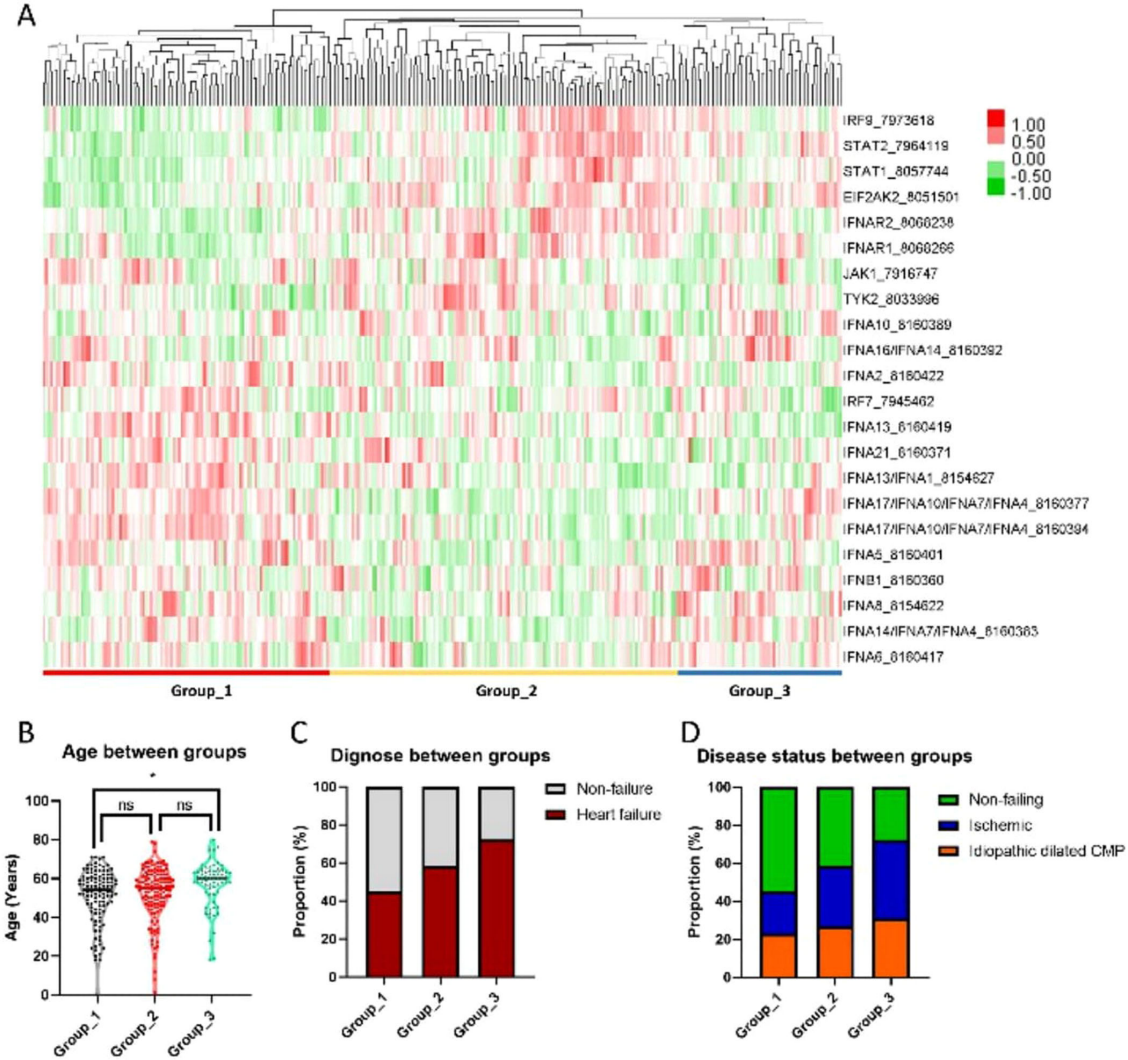
Comparison of groups by type I interferon related genes (TIIRGs) expression correlation (hierarchical clustering). According to TIIRG expression correlation (hierarchical clustering), patients in the discovery set are divided into three groups (A). Significant differences in age (B), EFrHF incidence (C), and pathological type (D) are observed among different groups.

**Figure 2:**
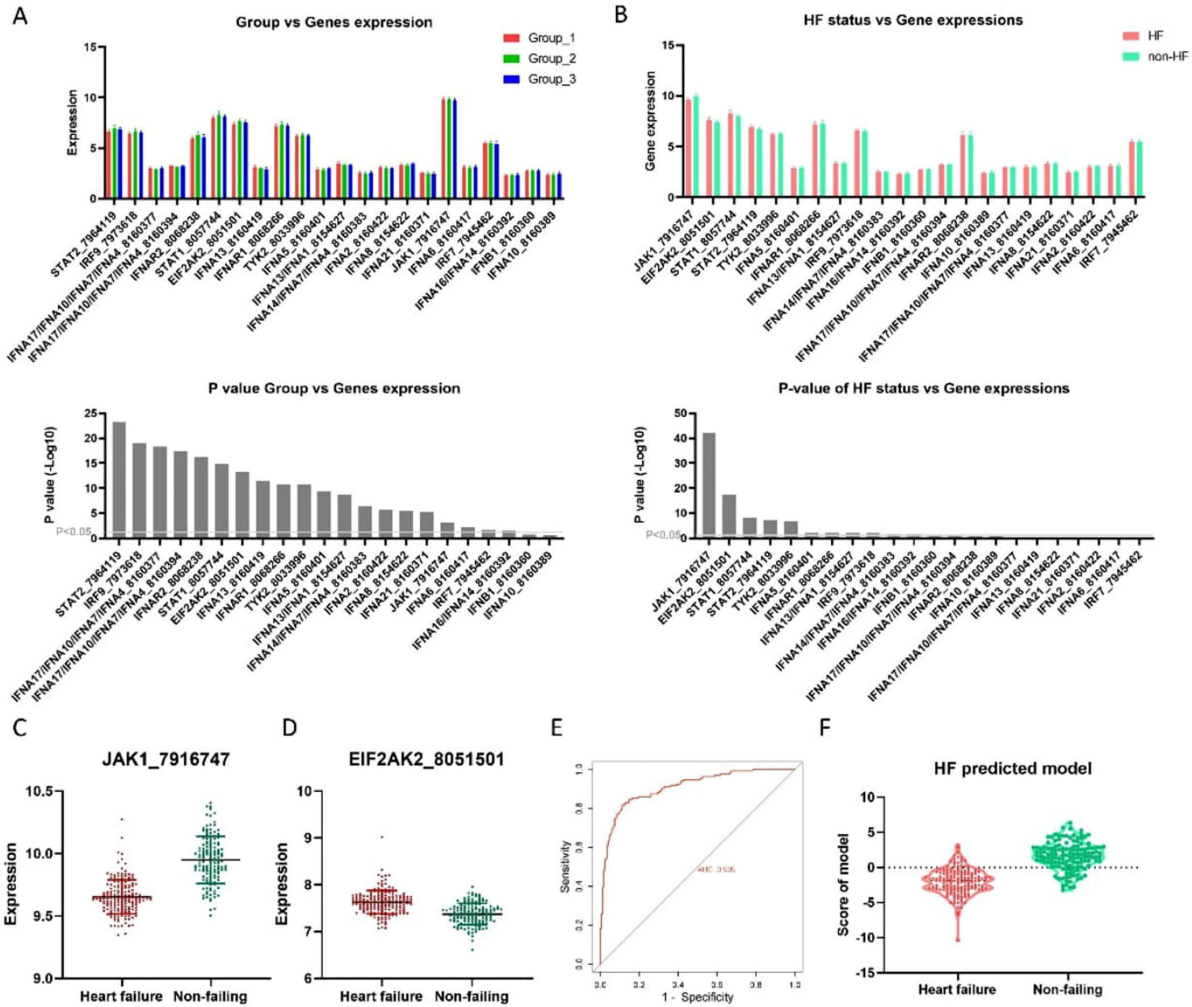
Comparative analysis of patients with EFrHF and nonheart failure (HF). Differences in gene expression were noted between different groups, and STAT2 was the gene with the most significant difference (A). The genes with the most significant differences between patients with EFrHF and non‐HF are JAK1, EIF2AK2, and STAT1 (B). In patients with EFrHF, JAK1 is significantly under‐expressed (C), whereas EIF2AK2 is significantly upregulated (D). An HF risk model with an AUC of 0.909 (E). When the risk score of the model is < 0, most patients with EFrHF can be screened out (F).

According to the correlation of TIIRG expression (hierarchical clustering), patients in the discovery set can be divided into three groups (Figure 1A), and differences in clinical characteristics were noted between each group (Table S1). Significant differences in age (Figure 1B), EFrHF incidence (Figure 1C), and pathological type (Figure 1D) were observed among different groups, indicating a close correlation between TIIRGs and EFrHF occurrence. Differences in gene expression were noted between different groups, and STAT2 was the gene with the most significant difference (Figure 2A).

### 3.2 Comparative Analysis of Patients With EFrHF and Non‐HF

Comparing the clinical data of patients with and without EFrHF, significant differences were observed regarding age and gender (Table S2). Patients over 57 years old and were males showed an increased risk of HF (odds ratio [OR] = 1.68, 95% confidence interval: 1.29–2.19 and OR = 1.52, 95% CI: 1.28–1.80). Furthermore, differences in gene expression were noted between the groups with different disease states, with a total of eight genes showing significant differences between patients with EFrHF and non‐HF (Table S3). The genes with the most significant differences between patients with EFrHF and non‐HF were JAK1, EIF2AK2, and STAT1 (Figure 2B). In patients with EFrHF, JAK1 was significantly under‐expressed (Figure 2C), whereas EIF2AK2 was significantly upregulated (Figure 2D). As with the quantitative method used in the validation set (GSE5406) after model construction, microarray data of transcriptional genomics was obtained from biological samples using the GPL96 platform gene expression chip. The results of these expression profiling were analyzed using the [HG‐U133A] Affymetrix Human Genome U133A Array corresponding to the GPL96 platform to obtain quantitative results of JAK1 and EIF2AK2 genes. Then an EFrHF risk model can be constructed by expressing JAK1 and EIF2AK2: risk score = JAK1 × 5.74 + EIF2AK2 × −7.53; AUC = 0.909 (Figure 2E). When the risk score of the model was < 0, most patients with EFrHF can be screened (Figure 2F).

### 3.3 Comparative Analysis of IDCM and ICM

**Figure 3:**
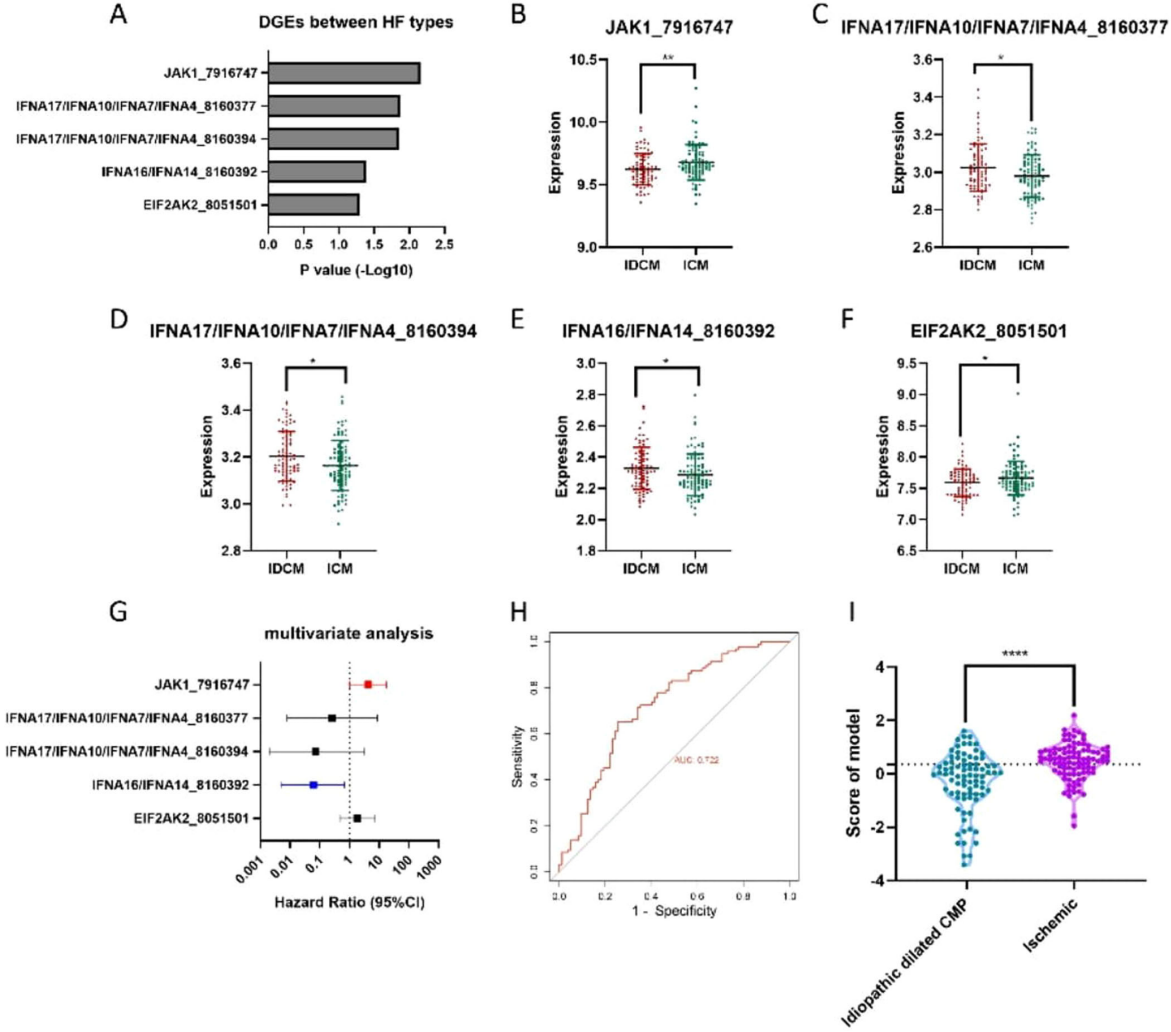
Comparative analysis of dilated cardiomyopathy (IDCM) and ischemic cardiomyopathy (ICM). Five genes with significant differences (A), among which JAK1 shows the most significant difference among different types of HF (B). Significant differences in IFNA17/IFNA10/IFNA7/IFNA4, IFNA16/IFNA14, and EIF2AK2 are observed between the two groups (C–F). Multivariate analysis shows that JAK1 and IFNA16/IFNA14 are independent risk factors between the two EFrHF types (G). To differentiate IDCM from ICM, a model can be constructed using JAK1 and IFNA16/IFNA14 (AUC = 0.722) (H). When the model value is < 0.359, most patients belong to IDCM (I).

IDCM and ICM are the two common EFrHF types. Their clinical diagnosis and treatment are different. Comparing the clinical data of the two showed a significant difference in age (Table S4). Comparing the gene expression among different EFrHF pathological types revealed significant differences in five genes (Figure 3A), among which JAK1 showed the most significant difference (Figure 3B). Additionally, significant differences were noted between the different types of IFNA17/IFNA10/IFNA7/IFNA4, IFNA16/IFNA14, and EIF2AK2, with both probes confirming the pathological differences between the two groups (Figure 3C–F). Multivariate analysis showed that JAK1 and IFNA16/IFNA14 were independent risk factors between the two pathological types (Figure 3G). To effectively distinguish between the two different types, a model can be constructed using JAK1 and IFNA16/IFNA14: predicted score = JAK1 × 3.06 + IFNA16/IFNA14 × 0.07–33.50; AUC = 0.722 (Figure 3H). When the model value was < 0.359, most patients belonged to IDCM (Figure 3I).

### 3.4 Age Analysis

**Figure 4:**
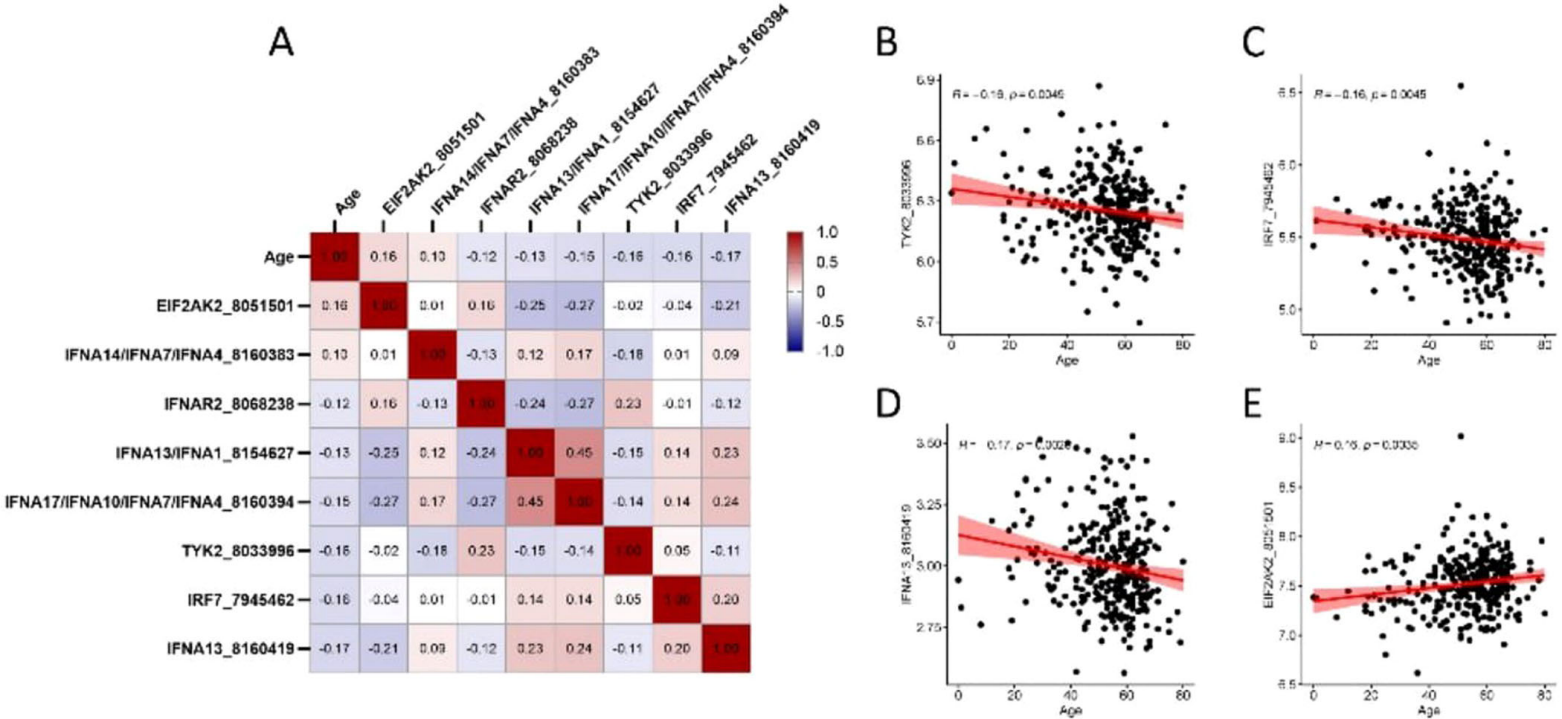
Age analysis. Eight gene expressions are correlated with increasing age, of which two are positively correlated and the other six are negatively correlated (A). The four significantly related genes are TYK2, IRF7, IFNA13, and EIF2AK2 (*R* > 0.15 or <−0.15, B–E).

The differences in age between the different TIIRG expression subgroups (Table S1, Figure 1) indicated that age was a significant correlation factor with TIIRGs. Moreover, age was a significant factor between EFrHF and non‐HF as well as between different pathological types; therefore, age‐related genes were specifically investigated. Eight gene expressions were correlated with increasing age, of which two were positively correlated and the other six were negatively correlated (Figure 4A). Among them, four significantly related genes were noted (*R* > 0.15 or <−0.15), including TYK2, IRF7, IFNA13, and EIF2AK2 (Figure 4B–E).

### 3.5 Differentiation of HF by JAK1 and EIF2AK2

**Figure 5:**
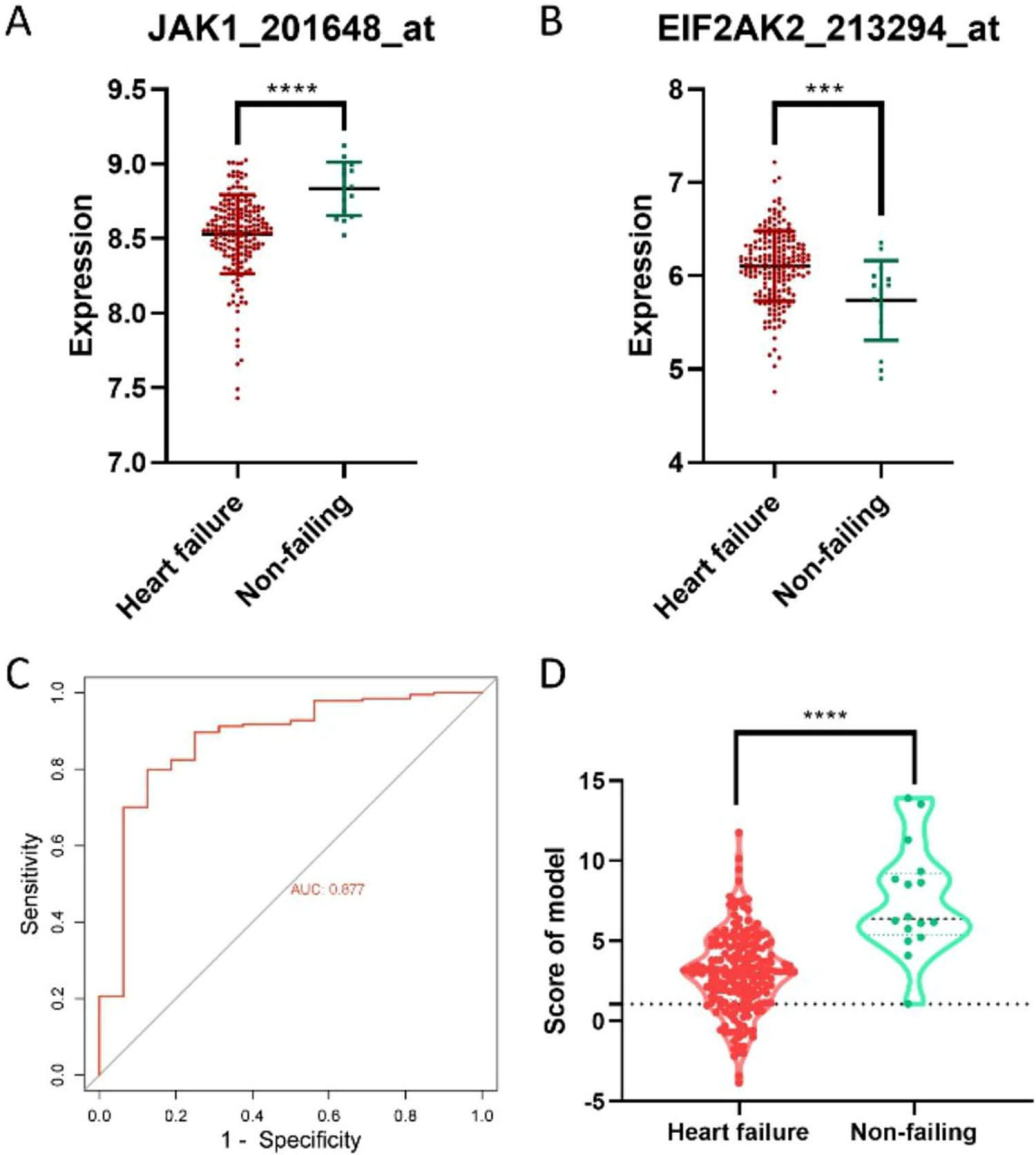
Differentiation of EFrHF by JAK1 and EIF2AK2. In HF tissues, JAK1 is significantly low expressed (A), whereas EIF2AK2 is significantly high expressed (B). A model of two genes can effectively distinguish between diseased and nondiseased individuals (AUC = 0.877, C). When the model score is < 1.08 (sensitivity = 1), 100% of patients with EFrHF will be identified (D).

Although JAK1 and EIF2AK2 expression was effective in predicting HF in the discovery set earlier (Figure 2), this finding only existed in a single data set and should be validated. Two gene expressions were observed in the validation set GSE5406. First, in HF tissues, JAK1 was significantly low expressed (Figure 5A), whereas EIF2AK2 was significantly high expressed (Figure 5B), which is consistent with previous findings. Furthermore, a model of two genes can effectively distinguish between diseased and nondiseased individuals (AUC = 0.877) (Figure 5C). When the model score was < 1.08 (sensitivity = 1), 100% of patients with EFrHF will be identified (Figure 5D).

## 4 Discussion

This study identified three different expression subtypes (groups 1–3) on the basis of the TIIRGs gene expression profile in the GEO data set. By comparing the clinical characteristics of different subtypes, significant differences in age, incidence, and causes of EFrHF were noted among the three groups. By comparing the TIIRG expression between different subtypes and between EFrHF and non‐HF tissues, significant differential genes, JAK1 and EIF2AK2, were identified. The prediction model constructed using these two genes can effectively distinguish between patients with EFrHF and non‐HF. We further compared the differential genes between IDCM and ICM and identified five different genes. These five genes had significant differences in expression between IDCM and ICM. Moreover, JAK1 and IFNA16/IFNA14 can be used for constructing models to accurately identify the two cardiomyopathies. TIIRG expression correlation (hierarchical clustering) showed three subgroups, with significant differences in the expression abundance and distribution of different TIIRGs in each group, which is related to the patient's age, EFrHF, and cause of EFrHF. Furthermore, our study identified that eight genes were positively correlated with age, with two positively correlated and the other six negatively correlated. Four significantly correlated genes were noted, including TYK2, IRF7, IFNA13, and EIF2AK2. Age is a traditional risk factor for EFrHF, and the impact of age on HF onset may be achieved through changes in gene expression [17]. However, currently, the underlying gene level mechanisms are not clear, and our research results suggest that TIIRGs can be involved. Therefore, clarifying the genes and mechanisms that play a major role in TIIRGs has important clinical significance. Comparing the expression differences of TIIRGs genes between patients with HF and non‐HF revealed eight differentially expressed TIIRGs, with JAK1, EIF2AK2, and STAT1 being the most significant. Previous studies reported that the JAK1/STAT signaling pathway plays a significant role in myocardial lesions [18]. This signaling pathway mediates the growth, survival, and apoptosis of cardiomyocytes and participates in cardiac angiogenesis regulation [19, 20]. Tyrosine protein kinases JAKs are receptor‐related proteins that regulate cell proliferation, differentiation, and apoptosis [21]. The STAT family is a significant JAK substrate, wherein STAT1 has proapoptotic and pro‐inflammatory effects, whereas STAT3 has antiapoptotic and anti‐inflammatory effects [22].

Negoro et al. proposed that JAK/STAT is activated during myocardial ischemia, thereby leading to increased STAT3 phosphorylation and increased expression of apoptosis‐related genes caspases‐3 and bax, indicating the antiapoptotic effect of the JAK/STAT signaling pathway [23]. Originally, eukaryotic translation initiation factor 2AK2 (EIF2AK2) was a pathogen recognition molecule [24]. To date, more studies have been conducted on its role in immune response and related diseases [25, 26]; however, its role in cardiovascular diseases remains unclear. Some studies have observed that EIF2AK2 overexpression promotes HeLa cell apoptosis, and EIF2AK2 selectively inhibits the transcription of SLE‐related histone genes, immune response genes, and TF genes [27]. This study observed that EIF2AK2 expression significantly increased in patients with HF and showed a trend of aging high expression. Therefore, whether EIF2AK2 also promoted cardiomyocyte apoptosis in patients with EFrHF deserves further study. The difference in TIIRG expression between IDCM and ICM shows that JAK1 and IFNA16/IFNA14 have a good differentiation effect on these two types of HF; particularly, the model constructed on the basis of these three genes can achieve an AUC of 0.722, and when the model value is < 0.359, most patients belonged to IDCM. This result has significant implications for clinical practice. Some patients with ICM have no typical clinical manifestations of myocardial ischemia in the past. When they are diagnosed with ICM, the heart cavity has expanded, and the ventricular wall has thinned. Differentiating from IDCM is challenging, and coronary angiography is frequently required. Coronary angiography is an invasive examination, with certain risks and high costs. If these two types of cardiomyopathies can be preliminarily identified through biomarker detection, coronary angiography can be more targeted. Moreover, our study noted that scores constructed on the basis of JAK1 and EIF2AK2 tissue expression levels have high differential diagnostic ability for HF. However, these scores are based on the expression levels of two genes in the tissue. Conducting tissue biopsy on patients with EFrHF in clinical practice is unrealistic; to clarify whether these two genes are expressed in the blood and whether their expression levels are consistent with those in the tissue, further research is needed. Meanwhile, the good discrimination ability of these scores for EFrHF suggests that these two genes can play a significant role in HF formation and progression. For a more accurate understanding of the mechanism of EFrHF and HF treatment, further research on the expression changes of these two genes during EFrHF and the relationship between downstream signaling pathways and EFrHF may be beneficial.

## 5 Limitations

This study had some limitations. First, this study was based on bioinformatics analysis of existing data, and the conclusions drawn were mainly based on statistical relationships. In the actual development process of EFrHF, the specific roles of the relevant genes in this study still require further cell and animal model research. Second, the results of this study were based on the results of genetic testing of myocardial tissues, which is a big example of clinical practice. Whether it can be converted into commonly used blood detection indicators in clinical needs further observation. Third, data in this study were mainly derived from populations in Europe and the United States, and whether it is suitable for patients from China is currently unclear. Furthermore, this study did not discriminate the impact of aging on final results.

## 6 Conclusion

TIIRG expression is closely related to the occurrence and pathological types of EFrHF, and it is significantly correlated with age. JAK1 and EIF2AK2 expression is closely related to the incidence and type of EFrHF, and the models constructed using JAK1 and EIF2AK2 can effectively diagnose EFrHF. Furthermore, EIF2AK2 expression is age related. Therefore, TIIRGs can serve as targets for the future prevention and treatment of EFrHF.

## Author Contributions

Concept and designed the study: Jianfeng Zhuo and Yan Zhong. Analyzed data and reviewing of the manuscript: Xiaojuan Luo and Sijie Qiu. Collected the data and helped in data analysis: Xiaojuan Luo and Yunyu Liang. Drafting of the manuscript: Yu Wu and Xiyu Zhang. Final editing and Guarantor of the manuscript: Sanjay Rastogi.

## Ethics Statement

The research conducted on human participants adhered to the ethical standards set forth by the institutional and/or national research committee of The Second Affiliated Hospital of Guangzhou University of Chinese Medicine and ethic committte of the "The Second Affiliated Hospital of Guangzhou University of Chinese Medicine" has approved the study. Additionally, it is imperative to adhere to the ethical guidelines outlined in the 1964 Helsinki Declaration and its subsequent revisions, or similar ethical standards. Moreover, written informed consent was obtained from all patients.

## Conflicts of Interest

The authors declare no conflicts of interest.

## Supporting information

Supporting information.

## Data Availability

The data sets used and/or analyzed during the current study are available from the corresponding author on reasonable request.
