# Supplementary material for: Heart Failure Is Closely Associated With the Expression Characteristics of Type I Interferon‐Related Genes
Source: Clin Cardiol. 2024 Dec 20;48(1):e70063. doi: 10.1002/clc.70063 (PMC11659751; doi:10.1002/clc.70063)
Supplement: Supplementary file 1 — Supporting information. [file CLC-48-e70063-s001.docx]

Supplementary Table 1: Clinical information of the identified groups.

| Classification |  | Group_1 | Group_2 | Group_3 | Sig. (P_value) |
| --- | --- | --- | --- | --- | --- |
| Number |  | n=113 | n=135 | n=65 |  |
| Age |  | 50.98 | 52.33 | 56.88 | 0.0167^*^ |
|  |  | 13.81 | 13.56 | 12.14 |  |
| Gender | female | 38 | 36 | 22 | 0.4084 |
|  | male | 75 | 99 | 43 |  |
| Heart_failure_status | Diagnosed | 51 | 79 | 47 | 0.0017^**^ |
|  | Healthy | 62 | 56 | 18 |  |
| Disease status | idiopathic dilated CM | 26 | 36 | 20 | 0.0091** |
|  | ischemic | 25 | 43 | 27 |  |
|  | non-failing | 62 | 56 | 18 |  |

^*^: P<0.05, ^**^: P<0.01.

Supplementary Table 2: Differences of age and gender between the patients with and without heart failure.

|  |  | Heart failure | non-Heart failure | P value | Odds Ratio for heart failure | 95% Confidence Interval | |
| --- | --- | --- | --- | --- | --- | --- | --- |
|  |  | n=177 | n=136 |  |  | Lower | Upper |
| Age | <=56 | 74 | 89 | 3.34361E-05^****^ | 0.64 | 0.52 | 0.79 |
|  | >57 | 103 | 47 |  | 1.68 | 1.29 | 2.19 |
| Gender | female | 33 | 63 | 1.40912E-07^****^ | 0.40 | 0.28 | 0.58 |
|  | male | 144 | 73 |  | 1.52 | 1.28 | 1.80 |

^****^: P<0.0001.

Supplementary Table 3: Differences in the gene expressions between the patients with and without heart failure.

|  | Sig. | Hazard Ratio (HR) | 95% C.I.for HR |  |
| --- | --- | --- | --- | --- |
|  |  |  | Lower | Upper |
| JAK1_7916747 | 9.7E-12^****^ | 2589.96 | 269.78 | 24863.84 |
| EIF2AK2_8051501 | 7.3E-11^****^ | 0.00 | 0.00 | 0.00 |
| STAT1_8057744 | 3.7E-02^*^ | 0.16 | 0.03 | 0.90 |
| STAT2_7964119 | 1.6E-03^**^ | 0.03 | 0.00 | 0.26 |
| TYK2_8033996 | 4.0E-01 | 3.02 | 0.23 | 39.58 |
| IFNA5_8160401 | 8.0E-01 | 1.42 | 0.09 | 21.24 |
| IFNAR1_8068266 | 1.7E-03^**^ | 29.57 | 3.57 | 244.69 |
| IFNA13/IFNA1_8154627 | 8.4E-01 | 1.27 | 0.12 | 14.07 |
| IRF9_7973618 | 2.9E-03^**^ | 37.68 | 3.45 | 411.21 |
| IFNA14/IFNA7/IFNA4_8160383 | 7.6E-03^**^ | 0.01 | 0.00 | 0.31 |
| IFNA16/IFNA14_8160392 | 8.0E-01 | 0.67 | 0.03 | 14.92 |
| IFNB1_8160360 | 6.7E-05^****^ | 0.00 | 0.00 | 0.03 |

^*^: P<0.05, ^**^: P<0.01, ^****^: P<0.0001.

Supplementary Table 4: Differences between the patients with different types of heart failure.

| Classification |  | IDCM | ICM | Sig. (P_value) |
| --- | --- | --- | --- | --- |
| Number |  | n=82 | n=95 |  |
| Age |  | 51.16 | 59.11 | 3.13347E-06^****^ |
|  |  | 13.98 | 7.39 |  |
| Gender | female | 19 | 14 | 0.150823035 |
|  | male | 63 | 81 |  |
| IFN-I Groups | 1 | 26 | 25 | 0.693656003 |
|  | 2 | 36 | 43 |  |
|  | 3 | 20 | 27 |  |

^****^: P<0.0001.
